# Infección diseminada por el virus del herpes simple en el embarazo

**DOI:** 10.7705/biomedica.7362

**Published:** 2024-11-06

**Authors:** Aída Oliveros, Paula Andrea Fonseca, Carlos Andrés Rodríguez, Javier Mauricio González

**Affiliations:** 1 Facultad de Medicina, Universidad Cooperativa de Colombia, Medellín, Colombia Universidad Cooperativa de Colombia Facultad de Medicina Universidad Cooperativa de Colombia Medellín Colombia; 2 Departamento de Farmacología y Toxicología, Universidad de Antioquia, Medellín, Colombia Universidad de Antioquia Departamento de Farmacología y Toxicología Universidad de Antioquia Medellín Colombia; 3 Departamento de Infectología, Hospital Manuel Uribe Ángel, Envigado, Colombia Departamento de Infectología Hospital Manuel Uribe Ángel Envigado Colombia

**Keywords:** herpes genital, herpes simple, embarazo, hepatitis, infecciones por herpesvirus, Herpes genitalis, herpes simplex, pregnancy, hepatitis, herpesvirus infections

## Abstract

Los virus herpes simple *(Herpes Simplex Virus,* HSV) de tipo 1 y 2 producen la infección de transmisión sexual más común en mujeres, con mayor incidencia en los países en desarrollo. Además de generar secuelas principalmente neurológicas en el recién nacido, cuando la primoinfección ocurre durante el periodo perinatal, puede diseminarse y producir gran morbimortalidad de la madre y el neonato.

A pesar de contarse con pruebas de laboratorio confiables, el diagnóstico de la infección por HSV durante el periodo perinatal es complejo, pues sus manifestaciones clínicas varían desde la forma diseminada hasta la asintomática o se pueden producir síntomas inespecíficos sin lesiones en piel o mucosas, por lo que es fundamental tener la sospecha clínica. Se presenta el caso de una madre con infección diseminada por HSV de tipo 2 que presentó hepatitis viral y cuyo neonato falleció, resaltándose la importancia de sospechar la infección por HSV en una mujer gestante febril con compromiso sistémico durante el periodo perinatal, aun en ausencia de brote.

## Abstract (en)

Herpes simplex virus (HSV) types 1 and 2 produce the most common sexually transmitted infection in women, with a higher incidence reported in developing countries. When the first infection occurs during the perinatal period, it can spread, resulting in high morbidity and mortality of the mother and child, in addition to mainly neurological sequelae in the newborn. Despite having reliable laboratory tests, the diagnosis of herpes simplex virus infection in this population is complex since the clinical presentation ranges from asymptomatic or with non-specific symptoms without lesions on the skin or mucous membranes. For this reason, a high clinical suspicion is necessary.

Here, we present the case of a mother with disseminated herpes simplex virus type 2 infection with viral hepatitis and fetal death, highlighting the importance of suspecting the diagnosis in febrile women with systemic compromise during the perinatal period, even in the absence of rash.

El herpes genital es una infección viral crónica y recurrente causada por dos tipos de virus del herpes simple *(Herpes Simplex Virus,* HSV): el HSV-1 y el HSV-2. La mayoría de casos de herpes genital son causados por el HSV-2 y se estima que el 11,9 % de las personas entre los 14 y los 49 años en Estados Unidos está infectada ^1^^-^^3^. En Suramérica, la prevalencia oscila entre el 20 y el 40 %; la población de mujeres en edad fértil es la más afectada, con grandes tasas de infección materno-fetal, principalmente, cuando la infección se adquiere en la segunda mitad del embarazo ^1^^,^^4^.

Se estima que el 10 % de las mujeres seronegativas para HSV-2 puede tener un compañero sexual seropositivo durante el embarazo, lo que aumenta el riesgo de adquirir la infección en el período de gestación ^5^.

Después de los pacientes inmunosuprimidos, las mujeres embarazadas con primoinfección por HSV son quienes más presentan la forma diseminada, con tasas de mortalidad entre el 38 y el 40 % ^1^^,^^5^^,^^6^. Esta diseminación también se asocia con aborto espontáneo, retraso en el crecimiento intrauterino, parto pretérmino e infección congénita en 30 a 50 % de los casos, y el riesgo que aumenta si la infección se contrae durante el tercer trimestre ^1^.

Las manifestaciones clínicas incluyen ampollas y ulceraciones en los genitales externos y el canal cervical, así como aumento de las secreciones vaginales, linfadenopatías y disuria u otros síntomas urinarios. Las manifestaciones sistémicas en las mujeres embarazadas pueden incluir fiebre, lesiones diseminadas en piel y complicaciones en otros órganos, como hepatitis, encefalitis y alteraciones hematológicas (trombocitopenia, leucopenia y coagulopatía), con una letalidad cercana al 50% ^1^^,^^5^^,^^6^. Dichas manifestaciones sistémicas pueden solaparse con las de otras enfermedades propias del embarazo, como el síndrome HELLP *(Hemolysis, Elevated Liver enzymes, Low Platelets),* el hígado graso del embarazo o la preeclampsia, lo cual dificulta un diagnóstico correcto y puede ocasionar graves complicaciones, como la falla hepática con necesidad de trasplante o la muerte ^7^^,^^8^.

Se presenta el caso de una mujer con infección diseminada por HSV-2, y se resaltan los riesgos para la madre y el producto de la concepción, y la importancia de la sospecha clínica y del tratamiento oportuno.

## Caso

Durante el cuarto pico de la pandemia de COVID-19, una mujer inmigrante de 20 años, con 34 semanas de gestación de su primer embarazo, asistió al servicio de urgencias por un cuadro de cinco días de evolución consistente en fiebre, malestar general, disnea, náuseas, dolor abdominal y aumento del flujo vaginal. El antecedente más relevante fue haber sufrido asma en la infancia. A su ingreso, se encontró febril, taquicárdica y taquipneica, sin desaturación de oxígeno ni hipotensión arterial; además, se observó flujo vaginal amarillo. El examen físico no mostró otros hallazgos relevantes.

Los exámenes de laboratorio en el momento del ingreso revelaron anemia con trombocitopenia y alcalosis respiratoria, sin otras alteraciones bioquímicas. Las pruebas de antígeno para COVID-19, la prueba rápida para HIV y las serologías para sífilis y toxoplasma fueron negativas. Se descartó bacteriemia e infección urinaria. En el examen directo del flujo vaginal, se observaron pseudomicelios y blastoconidias de levaduras, por lo que se recomendó tratamiento con clotrimazol en óvulos.

A las 48 horas de su ingreso, la madre continuaba con fiebre, taquicardia y taquipnea. La tomografía axial de tórax reveló derrame pleural bilateral con opacidades difusas en el lóbulo inferior izquierdo, lo que sugería un compromiso infeccioso, razón por la cual se administró ampicilina-sulbactam más azitromicina como tratamiento empírico. Los hemocultivos fueron negativos, al igual que la inmunoglobulina M (IgM) para leptospira y los anticuerpos antinucleares (ANAS). En la ecocardiografía se evidenciaron signos de hipertensión pulmonar, por lo cual se practicó una angiotomografía y se descartó tromboembolia pulmonar.

Al tercer día, la paciente presentó sangrado vaginal rutilante que requirió practicar una cesárea urgente. El recién nacido pesó 2.250 g y su talla fue de 45 cm; presentó buena adaptación neonatal.

**Figura 1:**
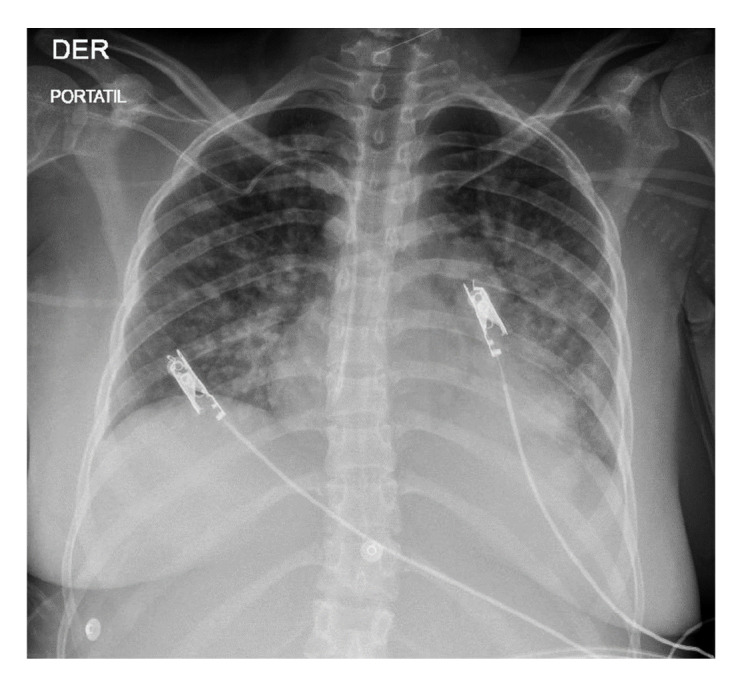
Radiografía portátil de tórax, vista anteroposterior, tomada en el séptimo día de ingreso. Se observan opacidades alveolares bilaterales con compromiso perihiliar difuso.

Al cuarto día, la madre continuaba febril y presentó choque con hipoxemia grave que requirió soporte vasopresor con noradrenalina. En los exámenes de laboratorio se encontró elevación de las transaminasas, proteinuria, gran aumento de la ferritina (≥ 2.000 ng/ml) e importante hipoalbuminemia; además, en los hemocultivos se encontró *Candida albicans,* por lo que recibió tratamiento con caspofungina. Asimismo, se observó hepatoesplenomegalia y progresión de las opacidades alveolares bilaterales (figura 1, cuadro 1).

**Cuadro 1:** Resultados de exámenes de laboratorios

Fecha	Día 1	Día 4	Día 8	Día 10	Día 19
Hemoglobina (g/dl)	9,9	11,8	7,7	7,2	-
Neutrófilos (1 x 10^3^)	3.500	4.800	3.820	8.100	-
Linfocitos (1 x 10^3^)	420	600	590	1.320	-
Plaquetas (1 x 10^3^)	64.000	84.000	62.000	193.000	-
PCR (mg/dl)	32	30	-	-	-
Creatinina (mg/dl)	0,7	-	-	0,57	
BUN (mg/dl)	6,5	-	-	-	-
TP/INR/s	11/0,7	-	-	16/1,55	24/2,3
TPT/s	37	-	-	44	48
ALT (U/L)	-	32	232	104	29
AST (U/L)	-	115	847	107	24
FA (U/L)	-	-	-	319	-
Bilirrubina total (mg/dl)	-	-	-	0,34	-
Bilirrubina indirecta (mg/dl)	-	-	-	0,05	-
Albúmina (g/dl)	-	-	1,8		-
LDH (U/L)	-	-	-	356	-
Proteína en 24 horas (g)	-	0,650			-
Ferritina (ng/ml)	-	-	>2000		-
Test de Tzank	-	-		Negativo	-
Dengue	Negativo	-	-	-	-
Leptospira	Negativo	-	-	-	-
PCR múltiple respiratoria	Negativo	-	-	-	-
Hemocultivos (15/01)	-	*Candida albicans*	-	-	-
ANAS, ENAS FR Negativos	-		-	-	-

BUN: nitrógeno ureico en sangre; PCR: proteína C reactiva; TP/INR: tiempo de protrombina (índice internacional normalizado); PTT: tiempo de tromboplastina; ALT: alanina aminotransferasa; AST: aspartato aminotransferasa; FA: fosfatasa alcalina; LDH: lactato deshidrogenasa

**Figura 2:**
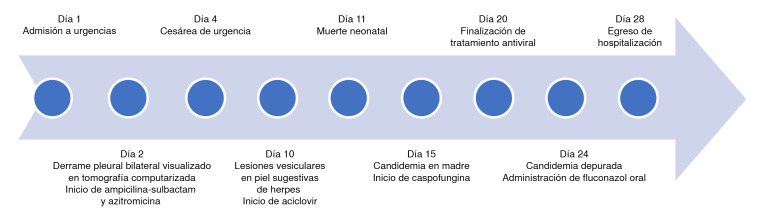
Línea de tiempo

Al séptimo día, la paciente continuaba febril y se evidenciaron lesiones vesiculares pequeñas en el tronco y en el miembro inferior izquierdo. Se practicó el test de Tzanck, cuyo resultado fue negativo. No obstante, ante la sospecha de herpes diseminado, se inició el tratamiento con aciclovir y se tomaron muestras para determinar la carga viral de HSV-1 y HSV-2 en suero. Los resultados de la PCR en tiempo real (qPCR) fueron positivos para HSV-2, con una carga viral de 7,55 x 10^6^ copias/ml (log 6,9). Por desabastecimiento de aciclovir intravenoso, se continuó el manejo con 5 mg/kg de ganciclovir cada 12 horas, hasta completar 10 días de tratamiento. Se obtuvo resolución de la fiebre y la fungemia cesó, por lo cual fue dada de alta después de 27 días de hospitalización (figura 2).

Por otra parte, el neonato desarrolló sepsis temprana con coagulopatía, que no mejoraron con el tratamiento médico. Falleció sin recibir medicamentos antivirales pues, en ese momento, no se tenía la sospecha de infección viral materna. La autopsia reveló cambios citopáticos sugestivos de herpes diseminado.

## Consideraciones éticas

Se obtuvo el consentimiento informado por escrito de la paciente para la publicación de este reporte de caso y las imágenes que lo acompañan.

## Discusión

La paciente desarrolló una infección diseminada por HSV-2, la cual se manifestó inicialmente con fiebre y un cuadro respiratorio agudo asociado con trombocitopenia, sin evidencia de lesiones vesiculares genitales o en otras regiones anatómicas; posteriormente, hubo elevación de las transaminasas séricas.

La ausencia de lesiones vesiculares típicas se ha reportado en casi la mitad de pacientes con herpes diseminado ^9^; esto constituye un reto para el cuerpo médico desde el servicio de urgencias hasta la unidad de cuidados intensivos y se asocia con retraso del diagnóstico. La infección diseminada puede causar sepsis viral, neumonitis, encefalitis y hepatitis, debido a la alteración de la inmunidad mediada por células, como ocurrió en el presente caso ^10^^,^^11^. Estas manifestaciones diversas se solapan con las de otras condiciones más frecuentes en las mujeres gestantes, como preeclampsia, síndrome HELLP e hígado graso agudo del embarazo, lo que dificulta aún más la sospecha de HSV diseminado ^7^^,^^12^.

En una serie de casos de pacientes embarazadas con infección por HSV, todas fallecieron, el 33,3 % por encefalitis y el 66,7 % por insuficiencia hepática. Como sucedió en el presente caso, ninguno de los productos de la concepción sobrevivió, lo cual indica la gran mortalidad fetal y neonatal que tiene esta enfermedad ^13^. Por lo tanto, es imperativo descartar esta infección en todo recién nacido que desarrolle sepsis temprana.

El HSV causa solo del 2 al 4 % de todas las hepatitis, pero tiene una gran morbimortalidad en inmunosuprimidos y mujeres gestantes, con una letalidad tan alta como del 40 % y falla hepática grave con necesidad de trasplante ^8^^,^^10^. En el presente caso, la paciente presentó hepatitis con coagulopatía y neumonitis, y solo se sospechó infección herpética cuando se observaron las lesiones en la piel. Según la revisión de Norvell *et al.,* el diagnóstico se sospecha solo en el 23 % de los casos de hepatitis por HSV y, en el 58 % de los pacientes, el diagnóstico solo se hace durante la autopsia ^14^.

Si bien el diagnóstico mediante pruebas de laboratorio es relativamente sencillo, la sensibilidad del test de Tzanck es solo del 52 % en las lesiones en mucosas ^15^. En cambio, las pruebas de biología molecular ofrecen excelentes sensibilidad y especificidad ^8^, aunque su disponibilidad puede ser limitada o se pueden requerir tiempos prolongados para obtener el resultado. Según esto, ante la sospecha clínica, se debe iniciar tratamiento empírico inmediato, como se hizo en el presente caso. La infección diseminada produce gran morbilidad en la madre y el producto de la concepción; no obstante, el tratamiento con aciclovir puede ser muy efectivo en las madres, como se observó en dos estudios: en uno, la mortalidad disminuyó del 88 al 51 % y, en el otro, del 67 al 20% ^14^^,^^16^. Infortunadamente, en el presente caso, el recién nacido falleció por sepsis neonatal sin recibir un tratamiento específico.

La infección por el virus del herpes simple hace parte del grupo TORCH de enfermedades infecciosas, que también incluye aquellas por *Toxoplasma gondii,* el virus de la rubéola y el citomegalovirus. El feto puede adquirir el virus mediante tres formas de transmisión principales: la placentaria (rara), durante el paso por el canal del parto (85 %) y la posnatal (10 %), cuando el recién nacido entra en contacto con lesiones por el HSV. Si bien los efectos teratogénicos son poco comunes, puede provocar anomalías congénitas; la tríada típica incluye lesiones cutáneas, anomalías del sistema nervioso central y alteraciones oculares ^17^.

La transmisión materno-infantil del HSV está influenciada por varios factores de riesgo, entre ellos:

1. el tipo de infección, en particular, si se trata del primer episodio;
2. el estado serológico de la madre, específicamente, si es seronegativa;
3. la tipificación del HSV en la lesión genital y su presencia en el momento del parto, y
4. el tiempo desde la ruptura de membranas ^17^^,^^18^.

Durante el período perinatal, el HSV puede provocar sepsis neonatal que aumenta la mortalidad y, generalmente, se manifiesta durante las primeras cuatro semanas de vida. Los recién nacidos con mayor riesgo son los prematuros y aquellos con bajo peso al nacer ^19^.

En el presente caso, la serología de la madre fue negativa para herpes 1 y 2 (IgG e IgM), lo que indicaba una infección primaria; el recién nacido desarrolló fiebre, coagulopatía y disfunción multiorgánica durante la primera semana de vida, sucumbiendo a una sepsis neonatal sin recibir tratamiento antiviral. El análisis *post mortem* reveló cambios citopáticos típicos. A pesar de que el parto por cesárea se asocia con un menor riesgo de infección neonatal por herpes, la transmisión perinatal se vio facilitada por la prematuridad, la falta de tratamiento materno para la infección primaria y una gran carga viral.

Existen varios factores de riesgo asociados con una mayor mortalidad en la infección neonatal por HSV ^20^. En este reporte, el neonato presentó enfermedad diseminada y no recibió tratamiento antiviral por retraso en el diagnóstico.

Por esto, ante la sospecha de infección neonatal por HSV, se recomiendan las pruebas moleculares y los cultivos de muestras de piel, mucosas o liquido cefaloraquideo según la sospecha clinica. Además, se debe practicar una punción lumbar para analizar el líquido cefalorraquídeo en busca de células mononucleares, pleocitosis, disminución de la concentración de glucosa y elevación de las proteínas. También, son necesarias la evaluación oftalmológica y la neurológica mediante resonancia magnética, incluso, en ausencia de afectación evidente del sistema nervioso central ^20^. En el presente caso, se practicó una punción lumbar y el análisis del líquido cefalorraquídeo reveló pleocitosis mononuclear leve (20 células/ ml), pero no se utilizó ninguna prueba molecular que ayudara a establecer el diagnóstico correcto ^21^.

La paciente presentó las características del síndrome hemofagocítico asociado con HSV-2: fiebre, hepatoesplenomegalia, alteración de los tiempos de coagulación, ferritina muy elevada e hipoalbuminemia, cuadro clínico que a menudo se confunde con el síndrome HELLP. En esta complicación se considera necesaria la terapia inmunosupresora ^22^, pero no se usó en el presente caso porque cursaba con candidemia secundaria.

Tanto en las guías de la *American Society of Obstetrics and Gynecology* como en las de la *European Society of Obstetrics and Gynecology*^23^^,^^24^, se recomienda el tratamiento antiviral en mujeres gestantes con HSV (primario o no) para reducir la gravedad y la duración de los síntomas, así como para eliminar las partículas virales. Generalmente, se administra aciclovir por vía oral, pero, en casos de enfermedad grave, se puede utilizar la vía intravenosa.

Para las infecciones adquiridas durante el primero o el segundo trimestre de gestación y para aquellas recurrentes, se recomienda una dosis de 400 mg de aciclovir por vía oral, tres veces al día durante cinco días. En todas las mujeres gestantes con antecedentes de herpes genital, se sugiere la inmunosupresión a partir de la semana 36 de para prevenir brotes recurrentes.

Si la infección se adquiere por primera vez en el tercer trimestre (a partir de las 28 semanas de gestación), se debe iniciar el tratamiento oral con 400 mg de aciclovir, tres veces al día, sin interrupción hasta el momento del parto, pues la terapia antiviral prenatal ha demostrado reducir el número de partículas virales en el canal del parto, las infecciones recurrentes y la necesidad de cesárea por herpes genital ^25^. La necesidad de la intervención cesárea depende de la presencia de lesiones en el canal vaginal o de la ruptura prematura de membranas.

Con el presente caso se resalta que, al final del embarazo, la infección diseminada por HSV puede manifestarse con fiebre (sin foco claro), encefalitis, hepatitis, neumonitis o síndrome hemofagocítico, por lo cual es importante incluirla en el diagnóstico diferencial de pacientes embarazadas febriles y con compromiso multisistémico, aun en ausencia de lesiones en piel o mucosas.

El retraso en el tratamiento puede costar la vida de la madre y el neonato, pues la mortalidad es significativamente menor en los casos tratados. Por esta razón, se recomienda iniciar el tratamiento empírico con aciclovir mientras se esperan los resultados de las pruebas confirmatorias ^5^. Un retardo en su inicio no solo empeora los resultados maternos, sino que aumenta el riesgo de transmisión vertical y muerte del producto de la concepción.
